# Oncocytic-type intraductal papillary mucinous neoplasm (IPMN)-derived invasive oncocytic pancreatic carcinoma with brain metastasis - a case report

**DOI:** 10.1186/1477-7819-10-138

**Published:** 2012-07-09

**Authors:** Kun-Chun Chiang, Chi-Chang Yu, Jim-Ray Chen, Yu-Ting Huang, Cheng-Cheng Huang, Chun-Nan Yeh, Chien-Sheng Tsai, Li-Wei Chen, Hsien-Cin Chen, Jun-Te Hsu, Cheng-Hsu Wang, Huang-Yang Chen

**Affiliations:** 1 General Surgery Department, Chang Gung Memorial Hospital, 222, Mai-Chin Road, Keelung, 204, Taiwan; 2 Graduate Institute of Clinical Medical Sciences, College of Medicine, Chang Gung University, 259 Wen-Hwa 1st Road, Kwei-Shan, Tao-Yuan, 333, Taiwan; 3 General Surgery Department, Chang Gung Memorial Hospital, 5, Fu-Hsing Street, Kwei-Shan, Taoyuan, 333, Taiwan; 4 Department of Pathology, Chang Gung Memorial Hospital, 222, Mai-Chin Road, Keelung, 204, Taiwan; 5 Department of Radiology, Chang Gung Memorial Hospital, 222, Mai-Chin Road, Keelung, 204, Taiwan; 6 Department of Radiation Oncology, Chang Gung Memorial Hospital, 222, Mai-Chin Road, Keelung, 204, Taiwan; 7 Department of Gastroenterolgy, Chang Gung Memorial Hospital, 222, Mai-Chin Road, Keelung, 204, Taiwan; 8 Department of Neurosurgery, Chang Gung Memorial Hospital, 222, Mai-Chin Road, Keelung, 204, Taiwan; 9 Department of Hematology and Oncology, Chang Gung Memorial Hospital, 222, Mai-Chin Road, Keelung, 204, Taiwan; 10 200, Lane 208, Jijin 1st Road, Keelung, 20445, Taiwan

**Keywords:** Brain metastasis, IPMN, Pancreatic cancer, Oncocytic IPMN

## Abstract

Pancreatic cancer is a lethal disease without effective treatments at present. It ranks as s as 4^th^ and 5^th^ in cancer-related mortality in the western countries and worldwide. Locally advanced pancreatic duct carcinoma (PDAC) and metastatic PDAC, usually found the metastases over liver, peritoneum, or lung, have been shown to be with dismal prognosis. Brain metastasis is a rare entity and most cases reported before were found post-mortem. Intraductal papillary mucinous neoplasms of the pancreas (IPMN) has been deemed as a precursor of PDAC with very slow progression rate. Here we reported a case diagnosed with IPMN-derived PDAC with brain metastasis. After surgeries for PDAC and brain metastasis, subsequent chemotherapy and radiotherapy were also given. One and half year after surgery, this patient is still living with good performance status, which may warrant individualization of therapeutic strategy for PDAC with only brain metastasis.

## Background

Pancreatic duct adenocarcinoma (PDAC) is a lethal human malignancies with a very poor prognosis. PDAC ranks as the eighth and ninth most common cause of cancer-related mortality worldwide for men and women, respectively [1]. The overall 5-year survival rate of patients with PDAC is only 1–4% approximately, which is possibly contributable to the aggressive characteristics of PCA [1]. PCA usually presents with early local spread and metastasis and resistance to traditional radiotherapy and systemic chemotherapies. At present, radical surgical resection remains the cornerstone of treatment. For advanced PDAC, the treatment strategy tends to be palliative.

Intraductal papillary mucinous neoplasms of the pancreas (IPMN) has been deemed as a precursor of PDAC with very slow progression rate. Here we reported a case diagnosed with IPMN-derived PDAC with brain metastasis. After aggressive surgeries for PDAC and brain metastasis and subsequent chemotherapy and radiotherapy, this patient is still l living well at the time of writing, which may encourages an aggressive treatment strategy against patients with PDAC with brain metastasis.

## Case presentation

**Figure 1:**
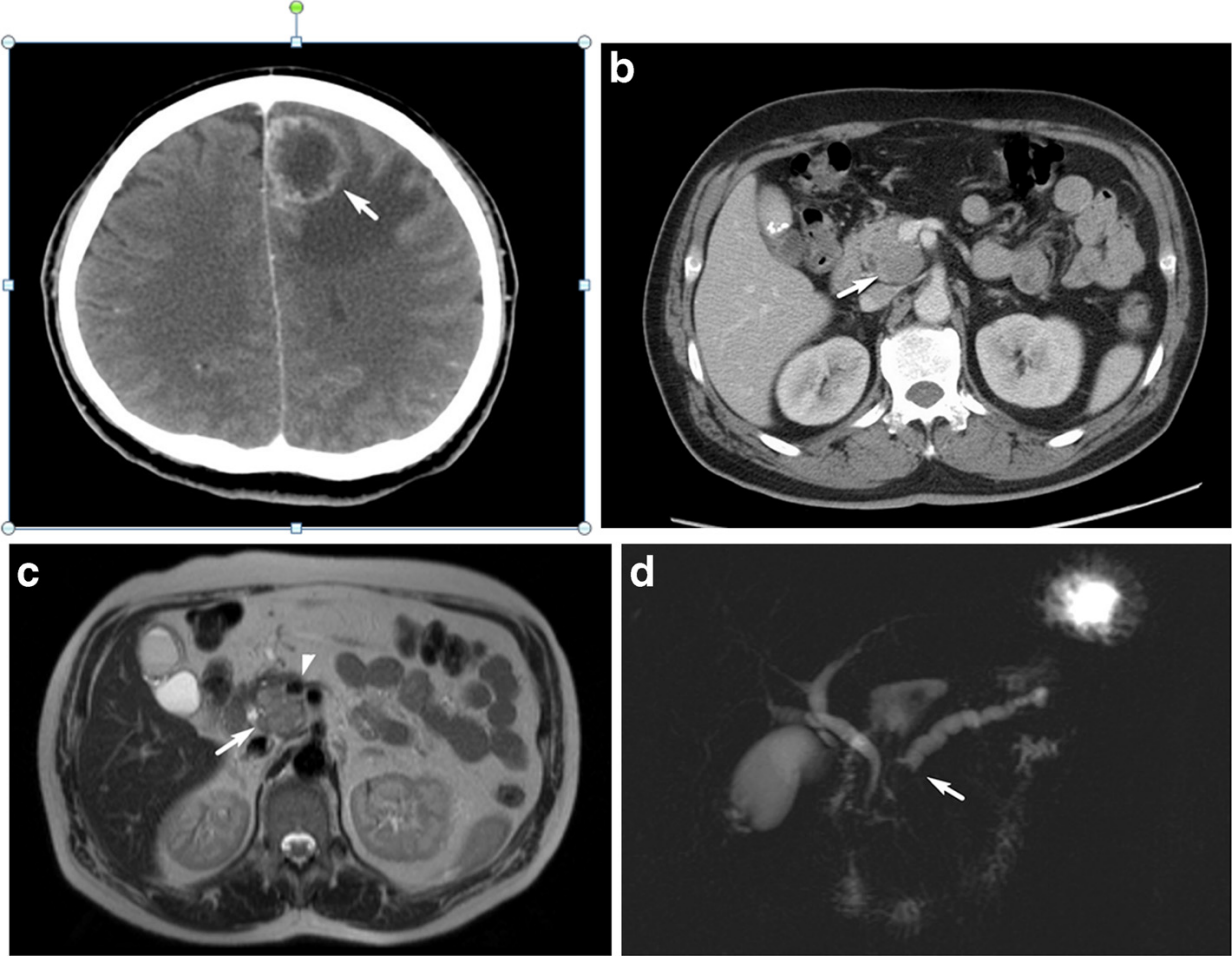
**Computed tomography (CT) and magnetic resonance (MR) imaging.** (**a**) Axial contrast-enhanced computed tomography of the brain shows a ring-enhancing lesion (arrow) in the left frontal lobe with perifocal edema. (**b**) Axial contrast-enhanced CT of abdomen shows a hypodense lesion (arrow) in the uncinate process of the pancreas with minimal encasement of the superior mesenteric vein. (**c**) 1 C T2-weighted image shows a mildly hyperintense mass (arrow) in the uncinate process of the pancreas with minimal encasement of the superior mesenteric vein (arrow head). (**d**) 1D MR cholangiopancreatography image shows dilatation of the pancreatic duct with abrupt termination (arrow). No dilatation of the biliary tree is noted.

A 54-year-old male patient with an unexceptional medical history complained of several episodes of right-sided weakness and tripping in November, 2009. After one episode of tumbling on January 2, 2010, he was brought to our emergency department by his family. Data from blood biochemistry and other blood tests were all within normal ranges. Due to the presence of neurologic symptoms and signs, a computed tomography (CT) scan of the patient’s brain was performed. The scan revealed a 2.9-cm low-density mass over the left frontal lobe, with peripheral rim enhancement and extensive perifocal edema reaching the corpus callosum, and crossing the midline (Figure 1A).

**Figure 2:**
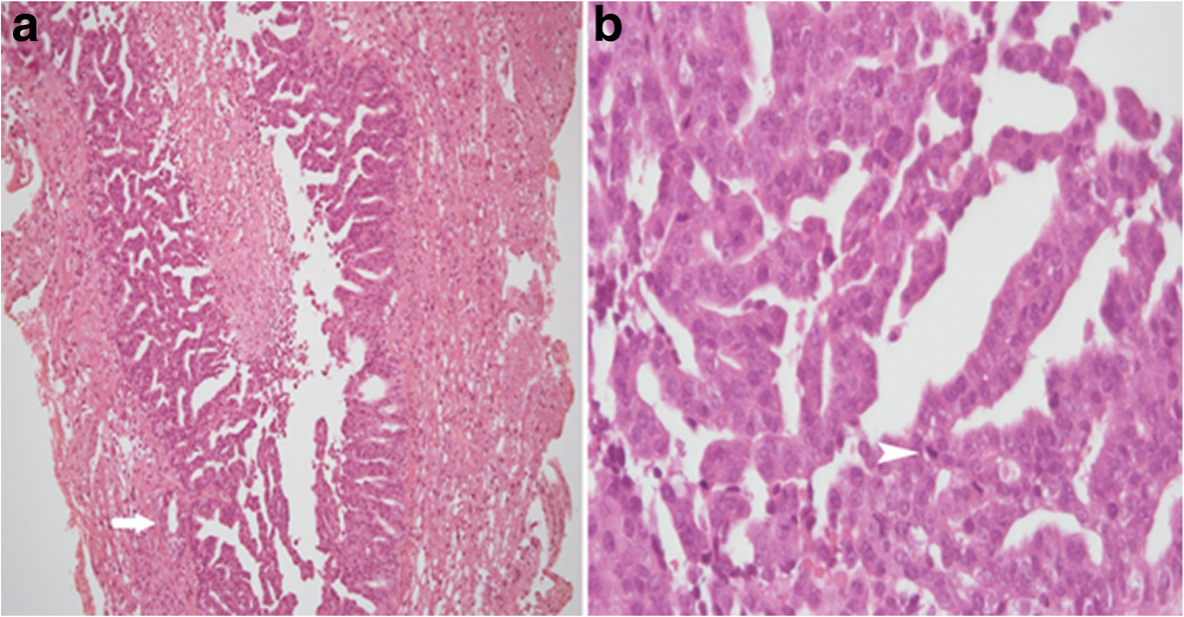
**Histological examination of the resected brain tumor.** (**a**) The brain tumor shows complex branching papillary structures with occasional tubular formation. (**b**) The tumor cells present large round nuclei with prominent nucleoli and abundant oncocytic cytoplasm. Mitoses are frequently seen.

A craniotomy was conducted on January 15, in the expectation of finding a brain abscess or tumor. During the operation, the cystic lesion was shown to contain no bacteria; the tumor was then resected subtotally due to its proximity to the motor area. The final pathology report indicated that the tumor was composed of branching complex papillae and occasional tubules (Figure 2A), with abundant oncocytic cytoplasm, large round nuclei with prominent nucleoli, and frequent mitoses (Figure 2B). Immunohistochemically, the tumor cells stained positively for cytokeratin 7, but were negative for cytokeratin 20, thyroid transcription factor 1 (TTF1), caudal-related homeobox transcription factor 2 (CDX2), and prostate-specific antigen, which indicated a metastatic rather than a primary brain tumor, which was unlikely to be derived from lung, gastrointestinal tract, or lung.

**Figure 3:**
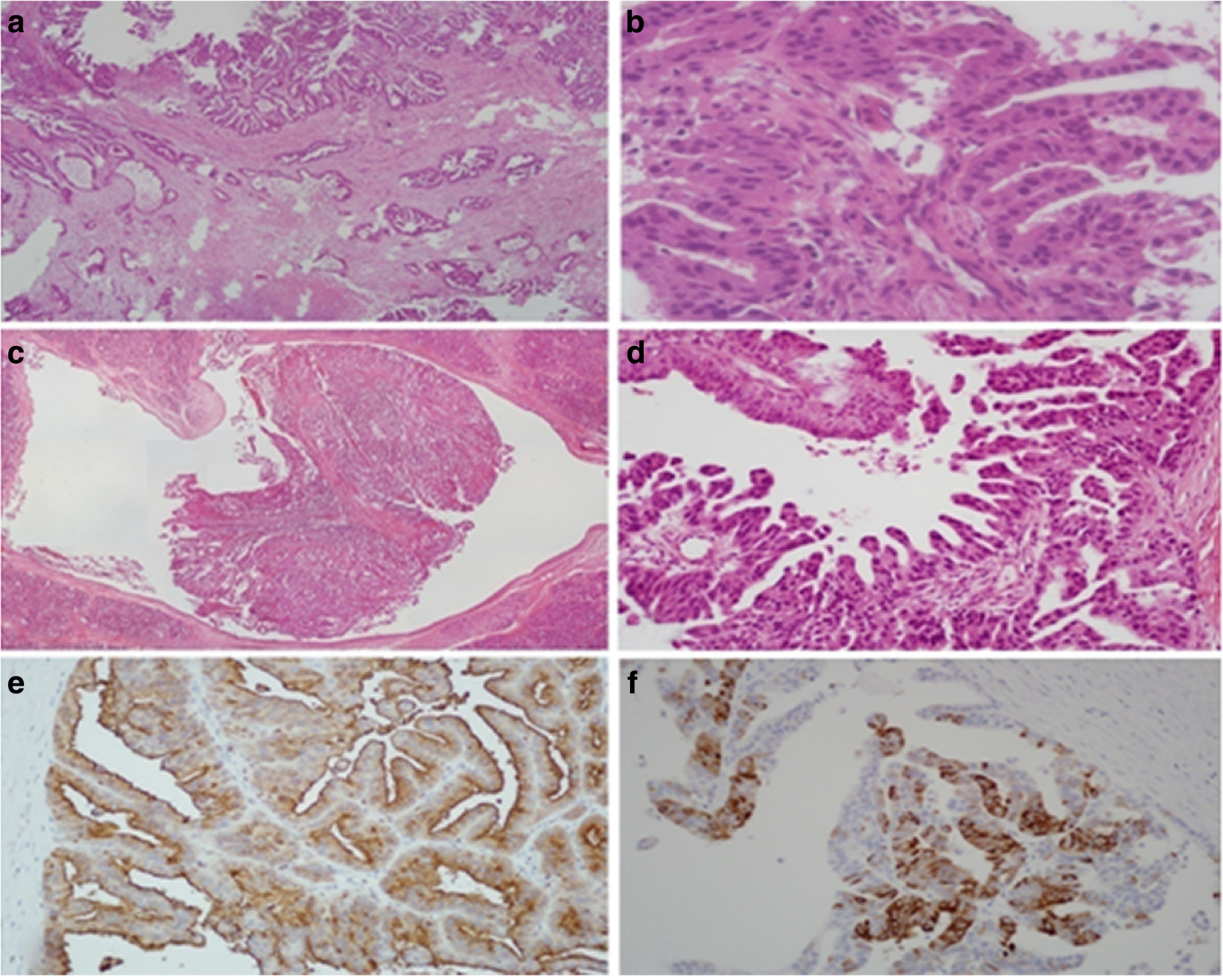
**Histological examination of the resected pancreatic tumor.** (**a**) The pancreatic head tumor shows complex branching papillary structures and infiltrating tubules. (**b**) The tumor cells contain mildly pleomorphic nuclei and oncocytic cytoplasm. (**c**) The dilated duct displays a polypoid tumor with complex arborizing papillary and tubular structures. (**d**) The tumor cells exhibit mildly pleomorphic nuclei and oncocytic cytoplasm. (**e**) Tumor cells are immunohistochemically positive for mucin protein, MUC1. (**f**) Mucin protein MUC5AC is positive for tumor cells under immunohistochemical stain.

A subsequent CT scan of chest and abdomen showed a 2.7-cm hypodense lesion in the uncinate process of the pancreas with pancreatic duct dilatation, suggesting pancreatic cancer (Figure 1B). Magnetic resonance cholangiopancreaticography (MRCP) (Figure 1C, D) was performed, revealing a 3.2-cm mass in the uncinate process of the pancreas with encasement of the superior mesenteric vein and pancreatic duct dilatation; the mass was radiologically staged as T4N0M0, according to the American Joint Committee on Cancer’s Cancer Staging Manual, 7^th^ editionA laparotomy was performed on the patient on February 26, 2010. The cancer was adhesive to the portal vein and superior mesentery artery, but was separable from them. The regional lymph nodes were grossly normal. A pylorus-preserving Whipple operation was then conducted, after which the patient recovered well and was discharged 2 weeks later. The resected pancreas showed a 4.5 × 4 × 2-cm infiltrative gray-white tu>mor, with some dilated ducts containing polypoid tumors. Histologically, the solid tumor showed diffusely infiltrating tubules and branching complex papillae composed of oncocytic cells having mildly pleomorphic nuclei (Figures 3A and 3B). In contrast, the dilated ducts contained polypoid tumors composed of oncocytic cells forming complex arborizing papillae and tubules (Figures 3C and 3D). Obvious mucin production was noted throughout the tumors. These tumors were immunoreactive with the mucin proteins MUC1 (Figure 3E) and MUC5AC (Figure 3F), but negative for MUC2, MUC6, and CDX2.

The immunophenotypes of the patient’s metastatic brain tumor were almost identical to those of the pancreatic tumors, except that they showed strong MUC6 staining. Additionally, both brain and pancreatic tumors showed a G12V (GGT → GTT) mutation in codon 12 of exon 1 of the *KRAS* gene [NCBI ID:3845] (data not shown). Taken together, these findings suggested a final diagnosis of oncocytic-type intraductal papillary mucinous neoplasm (IPMN)-derived invasive oncocytic carcinoma with brain metastasis [2,3], staged as T3N0M1.

After the operation, the patient received combined chemotherapy and radiotherapy (CCRT) consisting of irradiation of the whole brain with 3000 cGy, plus a boost of 1500 cGy divided into 5 doses; radiation of the pancreatic tumor bed with 5040 cGy divided into 28 doses, and chemotherapy based on cisplatin and 5-fluorouracil (5-FU).

During follow-up, no recurrence or metastasis was noted in the abdomen or elsewhere, with the exception of residual brain metastases, which presented with stationary status. The performance status of this patient remains good at the time of writing, 20 months after the operation.

## Discussion

Oncocytic-type intraductal papillary mucinous neoplasm (IPMN), first identified by Ohhashi *et al*. [4] in 1992 and first recognized by the World Health Organization (WHO) in 1996, is characterized by the presence of mucin-producing epithelial cells and cystic dilation in the pancreatic ducts. IPMNs represent more than one-third of all cystic neoplasms of the pancreas but less than 1% of all pancreatic tumors [5,6]. The malignancy potential of IPMNs is low; they are categorized histologically as low-grade, moderate-grade, high-grade, or invasive ductal carcinomas (IPMN-INV) [2,7]. Although IPMN is believed to progress slowly and to have a better prognosis after resection than pancreatic duct carcinoma (PDAC) [8,9], resected IPMNs have been found to include PDAC in 40% to 60% of cases [2,10-12]. On the basis of previous reports, the progression time from IPMN to PDAC varies, ranging from 5 to 20 years [10,13,14].

IPMN-derived PDACs have been divided into three distinctive subtypes: colloid carcinoma, oncocytic carcinoma, and tubular carcinoma [15]. Some studies have shown that IPMNs associated with invasive carcinomas result in better survival rates than conventional PDAC [16,17]. However, a recent matched-control study conducted by Yopp *et al*. showed that while IPMNs with invasive colloid carcinoma had a better prognosis than conventional PDAC [18], IPMNs with invasive tubular carcinoma had a prognosis comparable to that of conventional PDAC. Mino-Kenudson *et al*. further demonstrated that IPMNs with invasive colloid and oncocytic carcinoma had better survival rates than conventional PDAC, while IPMNs with invasive tubular carcinoma had a survival rate similar to that of conventional PDAC [15].

Here, we report a patient diagnosed with invasive oncocytic carcinoma derived from the oncocytic type of IPMN combined with sole brain metastasis, initially presenting with neurologic symptoms. As shown in a previous study, pancreatic cancer metastasizes predominantly to the liver and peritoneal cavity, as is usually found in locally advanced PDAC [19]. The incidence of brain metastases from pancreatic cancer is very low, with most cases being found post-mortem along with metastasis to places in addition to the brain [20,21]. Olson *et al*. reported the first ante-mortem case of PDAC with brain metastasis [22]. Recently, Marco *et al*. reported a case with PDAC staged as T3N1, in which the patient underwent R0 resection [radical resection ] and subsequent adjuvant chemoradiation. Two years later, brain metastasis occurred and gemcitabine-based chemotherapy was given [23]. To our knowledge, this is the first case of IPMN-derived PDAC with sole brain metastasis. Generally, the treatment for locally advanced PDAC or PDAC with metastasis is palliative, and the median survival time is within 10 months under current standard treatments (mostly gemcitamine-based chemotherapy) [24]. To the contrary, Vazquez *et al*. recently reported that patients with adrenal metastasis from pancreatic cancer seemed to be benefited by resection of metastatic and origin sites [25]. Patients with pancreatic cancer with one-sided liver metastasis used to undergo the Whipple operation and hepatectomy; however, no obvious improvement in survival has been reported. Some studies have shown that chemoradiotherapy could be more beneficial to patients than radiotherapy alone [26,27]. Used as a single agent to treat advanced PDAC, 5-FU has been studied extensively since the 1950s, with a range of objective response rates, varying from 0% to 67% [28]: 5-FU has also been combined with other agents, such as doxorubicin, cisplatin, cyclophosphamide, methotrexate, vincristine, and mitomycin, to treat advanced PADC. However, no significant survival benefit has been observed [29].

In the present case, our patient initially presented with neurologic symptoms and so was treated initially on the assumption that he was suffering from a brain abscess. After serial examinations, PDAC with brain metastasis was highly suspected. Although radical therapy for this situation was not feasible, due to the previous subtotal excision of the metastatic brain lesion and the fact that no other metastasis was found after a series of examinations, after discussion with his family, the Whipple operation was conducted and his final local stage was, surprisingly, T3N0. Subsequent radiotherapy and a combination of cisplatin and 5-FU chemotherapy were given and, at present, this patient is still alive with good performance status 2 years after surgery.

## Conclusions

On the basis of this case, we suggest that an aggressive therapeutic strategy may benefit some IPMN-derived PDAC patients presenting solely with brain metastasis. IPMN-derived PDAC with sole brain metastasis warrants individual treatment.

## Consent

This study has been approved by Chang Gung memorial hospital IRB board. The approved IRB number is 100-2977B. A copy of the approval of IRB is available for review by the Editor-in-Chief of this journal.

## Abbreviations

CCRT: Combined chemotherapy and radiotherapy; CDX2: Caudal-related homeobox transcription factor 2; CT: Computed tomography; 5-FU: 5-fluorouracil; IPMN: Intraductal papillary mucinous neoplasm; MR: Magnetic resonance; MRCP: Cholangiopancreaticography; PDAC: Pancreatic duct carcinoma; TTF1: Thyroid transcription factor 1; WHO: World Health Organization.

## Competing interest

The authors declare that they have no competing interests.

## Authors’ contribution

K-cC and C-cY wrote the manuscript, J-rC and C-cH carried out the pathological examination, C-nY, L-wC, J-tH helped wrote the manuscript, C-sT and H-cC participated in helping data collection, Y-tH carried out the image reading, C-hW and H-yC finalized the manuscript. All authors read and approved the final manuscript.

## Authors’ information

Huang-Yang Chen, Kun-Chun Chiang and Chi-Chang Yu are Co-first authors.
